# A Case of Cystic Degeneration of Both Ovaries, Following a Street Car Accident

**Published:** 1892-09

**Authors:** Jas. Kennedy

**Affiliations:** San Antonio, Texas


### DANIEL'S TEXAS MEDICAL JOURNAL.

ESTABLISHED JULY, 1885.

PUBLISHED MONTHLY.—SUBSCRIPTION $2.00 A YEAR.

Vol. VIII. AUSTIN, SEPTEMBER, 1892. No. 3.

### Original Contributions.

For Daniel's Texas Medical Journal:

### A CASE OF CYSTIC DEGENERATION OF BOTH OVARIES,

#### Following a Street Car Accident.

BY JAS. KENNEDY, M. D., SAN ANTONIO.

OPERATION—RECOVERY.

SINCE the introduction of the electrical system of street railways the casualties resulting from street car accidents have very greatly increased.

San Antonio, I believe, ranks third in point of mileage of electrical street railways among the cities of the United States, and has furnished her full quota of accidents and a great variety of injuries resulting therefrom.

The case which I am about to report resulted from an accident on one of our electrical street railway lines, and presents other features of interest in addition to the unusual character of the accident. The victim of this mishap was a young lady, aged about eighteen years, and of a robust constitution. She had enjoyed perfect health all her life, and up to the time of receiving the injury, had never suffered from any trouble of the womb or ovaries.

The way in which the accident occurred is said to have been as follows:

The car was being driven down grade at a reckless rate of speed and jumped the track at about the middle of the grade. The resistance of the cross-ties, which projected above the ground, and the momentum which the car possessed at the time it became derailed, caused it to swing round until at right angles with the track when it became arrested. The momentum not having been entirely expended, and the grade favoring, the car was almost overturned on its lower side; but, after dipping almost to the ground, it recoiled. The young lady, who had been sitting on the left hand side of the car prior to its derailment, when the car swung around transversely to the track, was then on the side highest on the grade; and when the car plunged broad-side and recoiled, she was thrown through the glass window of the opposite side of the car on the ground, above which the cross-ties projected, making a very hard and uneven surface upon which to fall.

It is impossible to state definitely just what part of the body received the greatest portion of the direct violence. Although it seems probable that the patient's left side and back came in contact with the ground first. She sustained considerable shock from the accident, but was scarcely rendered unconscious by it.

She was immediately picked up and placed in a carriage and driven to her home, which was some four blocks distant, to which place I was summoned. I saw her within an hour after the accident had happened, and found her lying upon a sofa, conscious, but suffering considerably from nervous shock. On making a careful examination for injuries, no external lesions of consequence were manifest. There were a few small scratches of the scalp,—one back of the ear and one or two on the back. In a day or two discolorations appeared on various parts of the trunk and extremities marking the site of the contusions which had been received.

The patient complained of headache and soreness all over the body. Pain seemed to be most marked over the abdomen, but as there was so much general soreness, I could not determine whether the tenderness over the abdomen was due to a bruising of the superficial structures; or to an injury to some of the abdominal or pelvic organs. After the general soreness had disappeared, pain localized in either pelvic fossa continued, and was most marked on the left side. When the patient attempted to assume the upright position she suffered great pain in her back in the lumbar region, and a dull, heavy and dragging pain in the pelvis which often became acute and lancinating. Suffering became so great that she could not assume the upright position at all, and was obliged to keep her bed. She suffered from headache and cold feet almost constantly. The bowels were constipated, and the appetite almost nil.

I placed the patient under the influence of chloroform and made an examination of the pelvic organs. The uterus was found to have suffered a postero-lateral version toward the left side. I corrected the displacement and caused the womb to be maintained in its normal position by means of tampons and rest in the recumbent posture for two weeks. After this operation she experienced great relief, but still suffered with pain in the back and pelvis, which was always most marked on the left side, and increased by assuming the upright position and by walking.

The accident occurred on the afternoon of December 23rd, and the operation of restoring the uterus to its normal position and relations was performed on January 26th following. I may here be allowed to state that the organ was found to be freely movable in the pelvis, and no change either in the size or contour of the ovaries could be detected at this time. For three weeks after the displacement had been corrected there was marked amelioration of the patient's suffering. But from this time on until the ovaries were removed the symptoms of suffering increased, and marked nervous disturbance became manifest.

During this latter period she suffered from pain in the back and over the region of the ovaries (most on left side), and radiating down the left thigh. Menstruation was very painful and markedly irregular. There was anorexia and obstinate constipation; the bowels often failing to move after the administration of copious enemata or full doses of cathartics. Constant and distressing cephalalgia, cold feet, insomnia, and later on, hysteria and hystero-epilepsy were added to the long train of distressing symptoms from which the patient suffered.

The hystero-epileptic attacks were almost invariably preceded by a feeling of general malaise and marked pallor of the countenance. These symptoms were often noticeable for twelve hours prior to an attack. The attack would commence by the occurrence of a sharp lancinating pain in the region of the left ovary, after which the patient would fall over in a swoon, which would last a few minutes, at the expiration of which the muscles would take on spasmodic contractions. The extremities were first affected in this manner, and afterwards the spinal muscles. Marked opisthotonos always existed during the greater part of the attack. The pupils were always widely dilated.

When muscular spasm would subside, delirium, which followed the brief spell of coma, continued, and at her last attack fourteen hours elapsed before normal consciousness was restored.

The treatment which I employed in my effort to effect a cure in this case embraced posture, rest, anodynes and hypnotics. Emollient stimulant and counter-irritant applications to the abdomen were tried in their turn but without effect. Hot douching of the vagina, applications of tincture of iodine to the fornix vaginæ and glycerine tampons were among the local measures practiced with a view of relieving the patient, but to no avail.

After six months of constant suffering, the patient demanded relief at any cost. The treatment followed so far had failed to effect a cure, or even permanently benefit the patient, and I had exhausted every therapeutic resource that had been suggested by consultants who had seen the case with me, yet her condition had grown steadily worse.

I again examined the patient and found the uterus occupying its normal position. The left ovary was enlarged to about three or four times its normal size, while the right ovary did not seem to be increased in size. Both were exquisitely sensitive.

After this examination I informed the patient of the condition in which I found her reproductive organs, and told her that in my opinion the removal of the diseased ovary was the only means of cure that had not been tried in her case, and that promised permanent results.

The gravity and danger, together with the nature and probable consequences of the operation, were fully explained to the patient and her family, after which an operation were insisted upon by them.

Consultations with several physicians were held, and while we were practically agreed as to the patient's condition, the necessity for operation was disputed by one of my colleagues who has achieved renown, both as a surgeon and gynecologist. He could suggest no substitute for the operation, however, as I had already exhausted his fertile therapeutic resources in previous consultations on this case. In the face of the patient's history and condition of constant suffering, he advised delay. His objection to the operation, as I understood, was based upon a sentiment of aversion to the emasculation of young females, which sentiment is indeed laudable, so long as we confine its application to those cases where the indications for operation are not clear and positive. However, the majority favored the operation, and ovariotomy was accordingly performed.

Before proceeding further, I wish to state that I explained to both the patient and her relatives that it might be found necessary to remove both ovaries, and informed them of the consequences that naturally follow this radical procedure.

OPERATION.—An incision, two and a half inches in length, was made through the abdominal walls (which contained much adipose), in the median line between the pubes and umbilicus. The fascia and peritoneum were divided on a grooved director. The tumor was seized between the thumb and fingers, and drawn out through the wound, tied off, and excised. The right ovary was also enlarged and diseased, and was treated in a similar manner.

The abdomen was flushed with sterilized water, the wound closed with silver wire sutures, which included the peritoneum, after a glass drainage tube had been introduced, through which were passed strips of iodoform gauze, dipping into Douglas' cul-de-sac and out through the incision at its lower angle.

The most rigid asepsis and antisepsis were practiced throughout.

The left ovary was nearly four times its normal size, and three-fourths of its substance was occupied by a cyst with fluid contents which appeared translucent when held up to the light. The right ovary was somewhat enlarged and elongated. About one-fourth of its substance was occupied by a cyst. The tubes were thickened and congested.

From the right tube was suspended a small cyst, attached by a long pedicle of thread like character.

The dressings were changed on the third day, and the tube removed on the fourth day. The bowels moved on the fifth day, and have been regular in their functions ever since.

The stitches were removed on the sixth day. The patient sat up on the thirteenth day, and went out for a carriage ride on the fifteenth day.

The temperature ranged from 99° F. to 101.8° F.

On the seventeenth day, an abscess of the abdominal walls opened in the cicatrix, and evacuated an ounce or two of pus.

I will here mention that the breaking of the abscess was the first intimation of its existence. The wound had entirely healed, and the tissues were normal in appearance. The temperature became normal, and so remained. It was thus clearly evident that the elevation of temperature to 101° F., as late as the fifteenth day, was due to the existence of this abscess.

The operation was performed on the fourth day of June, and since that time all of the distressing phenomena from which the patient suffered have been entirely absent.

### REMARKS.

There are two points that I wish to emphasize particularly, in connection with this report. One is, that in accidents of this character it is our imperative duty to ascertain whether or not the pelvic organs have sustained any damage.

We are liable to be misled by allowing too much for the pain due to bruising of the external or superficial structures, and a pain in the back may be attributed to a contusion of the spinal muscles or of the spine itself, when in reality it may be the result of a displacement of the uterus.

By making an examination of the pelvic organs in a careful manner, we are not likely to do any harm; and if a displacement of the uterus or prolapsus of the ovary exists, the early restoration of the organ to its natural position is of decided benefit to the patient.

The other point I wish to call attention to, is the question of prognosis.

It goes without saying that the patient, her family and friends, are anxious, in such cases, to know what the probable final result of the injury will be, and as all railway companies are chartered corporations, and consequently liable for damages, in all cases of injury resulting from negligence on the part of either themselves, their agents or employes, the determination of the extent of the injury or injuries so received, the amount and duration of the disability resulting therefrom, and last but not least, whether the patient so injured will completely recover, is a matter of great importance to all parties concerned.

To my mind, there is no class of cases in which the prognosis is more uncertain than in injuries to the pelvic viscera. It is easy enough to determine whether any disturbance of their relations has been occasioned by the violence,—that is, to say whether the uterus or the ovaries have suffered any displacement; but it is not possible for us to tell just what effect this disturbance of relations may eventuate in, even though the organs were promptly restored to their natural position. In other words, we cannot tell whether the ligaments were simply stretched or torn, whether the uterus, ovaries or tubes suffered contusion.

Nor can we tell what the effect of even a slight contusion of the ovary may result in. Even in a simple displacement of the uterus, grave results may follow at a remote period, although the organ may have been promptly replaced. The ligaments may remain lax from the over-stretching to which they were subjected, and a version may recur, or a prolapse ensue, upon a slight provocation, with all its attendant pathological and clinical phenomena.

The mere subsidence of pain after an injury of the kind described, does not justify the assumption that the patient will pass on to a complete recovery. A sub-acute or chronic inflammatory process may supervene upon the decline of the acute, and go on for a long time insidiously, without occasioning the patient very much distress, and finally result in a pathological condition necessitating the removal of the ovary.

I think I have said sufficient on this point to impress the fact that we should feel it our duty to inform our patients, and also those who are more or less immediately interested, as to what the *possibilities* are in injuries of this character, and at the same time to be very conservative in giving our opinion as to the *probable* outcome in any given case.

July, 1892.

